# Methyl 1-phenyl-3-*p*-tolyl-1,9b-di­hydro-3*H*-chromeno[4,3-*c*]isoxazole-3a(4*H*)-carboxyl­ate

**DOI:** 10.1107/S1600536814002438

**Published:** 2014-02-12

**Authors:** B. Raghuvarman, J. Srinivasan, M. Bakthadoss, S. Aravindhan

**Affiliations:** a Department of Physics, Presidency College (Autonomous), Chennai 600 005, India; b Department of Organic Chemistry, University of Madras, Guindy Campus, Chennai 600 025, India

## Abstract

In the title compound, C_25_H_23_NO_4_, the pyran ring of the chroman moiety has an envelope conformation with the methyl­ene C atom as the flap. The isoxazole ring has a twist conformation on the O—C bond. The dihedral angle between their mean planes is 57.87 (9)°. The attached phenyl and benzene rings are twisted away from its mean plane by 56.19 (10) and 50.57 (10)°, respectively. These two rings are normal to each other, subtending a dihedral angle of 89.2 (1)°. In the crystal, there are no classical hydrogen bonds; the mol­ecules are linked *via* C—H⋯π inter­actions, forming a two-dimensional network lying parallel to (10-1).

## Related literature   

For the biological activity of isoxazoline derivatives, see: Kozikowski (1984 ▶); Howe & Shelton (1990 ▶); Bakthadoss & Murugan (2010 ▶). For the synthesis of chromenoisoxazolidines by intra­molecular 1,3-dipolar cyclo­additions, see: Bakthadoss & Murugan (2010 ▶). For puckering parameters, see: Cremer & Pople (1975 ▶). For asymmetry parameters, see: Nardelli (1983 ▶). For standard bond lengths, see: Allen *et al.* (1987 ▶).
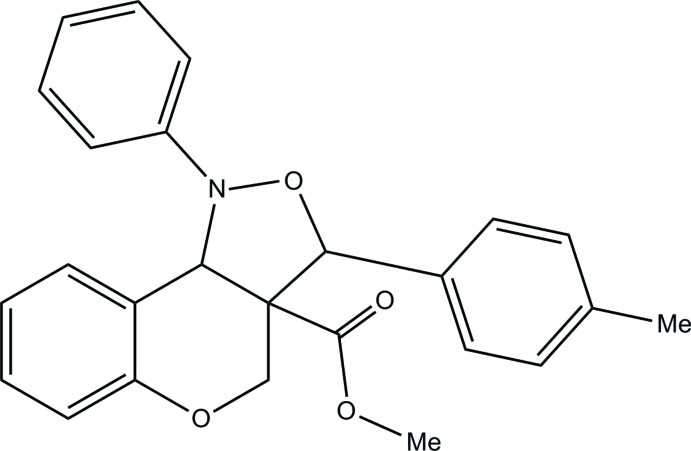


## Experimental   

### 

#### Crystal data  

C_25_H_23_NO_4_
*M* *_r_* = 401.44
Monoclinic, 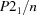

*a* = 14.0674 (7) Å
*b* = 7.8105 (4) Å
*c* = 19.7680 (9) Å
β = 110.456 (3)°
*V* = 2035.01 (17) Å^3^
*Z* = 4
Mo *K*α radiation
μ = 0.09 mm^−1^
*T* = 293 K
0.22 × 0.19 × 0.17 mm

#### Data collection  

Bruker SMART APEXII CCD diffractometer
Absorption correction: multi-scan (*SADABS*; Bruker, 2008 ▶) *T*_min_ = 0.981, *T*_max_ = 0.985
18922 measured reflections
5086 independent reflections
3415 reflections with *I* > 2σ(*I*)
*R*_int_ = 0.035

#### Refinement  

*R*[*F* ^2^ > 2σ(*F* ^2^)] = 0.051
*wR*(*F* ^2^) = 0.151
*S* = 1.02
5086 reflections
273 parameters
H-atom parameters constrained
Δρ_max_ = 0.35 e Å^−3^
Δρ_min_ = −0.39 e Å^−3^

### 

Data collection: *APEX2* (Bruker, 2008 ▶); cell refinement: *SAINT* (Bruker, 2008 ▶); data reduction: *SAINT*; program(s) used to solve structure: *SHELXS97* (Sheldrick, 2008 ▶); program(s) used to refine structure: *SHELXL97* (Sheldrick, 2008 ▶); molecular graphics: *ORTEP-3 for Windows* (Farrugia, 2012 ▶); software used to prepare material for publication: *SHELXL97* and *PLATON* (Spek, 2009 ▶).

## Supplementary Material

Crystal structure: contains datablock(s) global, I. DOI: 10.1107/S1600536814002438/su2676sup1.cif

Structure factors: contains datablock(s) I. DOI: 10.1107/S1600536814002438/su2676Isup2.hkl

Supporting information file. DOI: 10.1107/S1600536814002438/su2676Isup3.cml

CCDC reference:

Additional supporting information:  crystallographic information; 3D view; checkCIF report

## Figures and Tables

**Table 1:** Hydrogen-bond geometry (Å, °) *Cg*3 and *Cg*5 are the centroids of rings C1–C6 and C17–C22, respectively.

*D*—H⋯*A*	*D*—H	H⋯*A*	*D*⋯*A*	*D*—H⋯*A*
C2—H2⋯*Cg*5^i^	0.93	2.91	3.757 (2)	151
C25—H25*B*⋯*Cg*3^ii^	0.96	2.71	3.450 (3)	134

Symmetry codes: (i) 
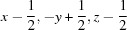
; (ii) 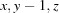.

**Fig. 1.**
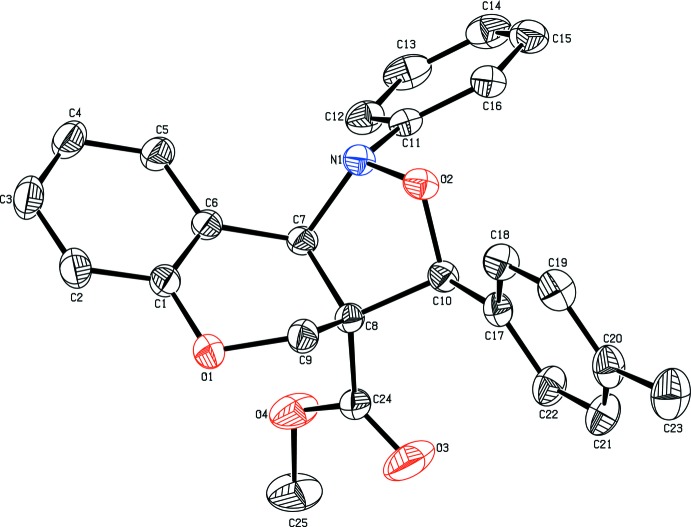
The molecular structure of the title molecule, with atom labelling. The displacement ellipsoids are drawn at 30% probability level. the H atoms have been omitted for clarity.

## supplementary crystallographic information

### 1. Comment

Isoxazoline derivatives have been shown to be efficient precursors for the preparation of many synthetic intermediates including γ-amino alcohols and β-hydroxy ketones (Kozikowski, 1984). They display interesting biological properties such as herbicidal, plant growth regulators and antitumour activities (Howe & Shelton, 1990). The title compound in which a chromane and isoxazole ring are fused was synthesized by (Bakthadoss & Murugan, 2010), and we report herein on its crystal structure.

The molecular structure of the title molecule is shown in Fig. 1. The bond lengths (Allen *et al.*, 1987) and bond angles are normal. The pyran ring (O1/C1/C6-C9) of the chromane moiety adopts an envelope conformation with atom C9 as the flap; puckering parameters (Cremer & Pople, 1975) and asymmetry parameters (Nardelli,1983) are: q_2_=0.405 (2) Å, q_3_ = -0.247 (2) Å, φ_2_ = 123.9 (3)° and Δ_s_(C9)= 6.26 (2)°, respectively.

The isoxazole ring (N1/C7/C8/C10/O2) has a twist conformation on bond O2-C10. The attached aromatic rings (C11—C16 and C17—C22) are twisted away from its mean plane by 56.19 (10)° and 50.57 (10)°, respectively. The two aromatic rings are normal to each other with a dihedral angle of 89.2 (1) °.

The carboxylate group assumes an extended conformation which can be seen from the torsion angle C8—C24—O4—C25 = -179.18 (19)°.

In the crystal, there are no classical hydrogen bonds. The molecules are linked via C—H···π interactions (Table 1), forming a two-dimensional network lying parallel to plane (1 0 -1).

### 2. Experimental

The title compound was synthesized according to the published procedure (Bakthadoss & Murugan, 2010). A mixture of (E)- methyl 2-((2-formylphenoxy)methyl)-3-*p*-tolylacrylate (2 mmol, 0.60 g) and *N*-phenylhydroxylamine (3 mmol, 0.33 g) in ethanol (10 ml) was refluxed for 6 h. After the completion of the reaction, as indicated by TLC, the reaction mixture was concentrated under reduced pressure and the resulting crude mass was diluted with water (15 ml) and extracted with ethyl acetate (3 × 15 ml). The organic layers were combined and washed with brine (3 × 15 ml) and dried over anhydrous Na_2_SO_4_, and the solvent was removed under reduced pressure. The crude mass was purified by column chromatography on silica gel (Acme 100–200 mesh), using ethyl acetate-hexane (0.5: 9.5) to afford the pure compound as a colourless solid in 92% yield. Colourless block-like crystals were obtained by slow evaporation of a solution in ethyl acetate-hexane (0.5: 9.5).

### 3. Refinement

N and C-bound H atoms were positioned geometrically and allowed to ride on their parent atoms: N-H = 0. \%A, C–H = 0.93–0.98 Å, with *U*_iso_(H) =1.5*U*_eq_(C-methyl) and = 1.2*U*_eq_(C) for other H atoms.

### Crystal data

**Table:**

C_25_H_23_NO_4_	*F*(000) = 848
*M**_r_* = 401.44	*D*_x_ = 1.310 Mg m^−^^3^
Monoclinic, *P*2_1_/*n*	Mo *K*α radiation, λ = 0.71073 Å
Hall symbol: -P 2yn	Cell parameters from 3415 reflections
*a* = 14.0674 (7) Å	θ = 1.6–28.4°
*b* = 7.8105 (4) Å	µ = 0.09 mm^−^^1^
*c* = 19.7680 (9) Å	*T* = 293 K
β = 110.456 (3)°	Block, colourless
*V* = 2035.01 (17) Å^3^	0.22 × 0.19 × 0.17 mm
*Z* = 4	

### Data collection

**Table:**

Bruker SMART APEXII CCD diffractometer	5086 independent reflections
Radiation source: fine-focus sealed tube	3415 reflections with *I* > 2σ(*I*)
Graphite monochromator	*R*_int_ = 0.035
ω and φ scans	θ_max_ = 28.4°, θ_min_ = 1.6°
Absorption correction: multi-scan (*SADABS*; Bruker, 2008)	*h* = −18→16
*T*_min_ = 0.981, *T*_max_ = 0.985	*k* = −10→8
18922 measured reflections	*l* = −26→26

### Refinement

**Table:**

Refinement on *F*^2^	Primary atom site location: structure-invariant direct methods
Least-squares matrix: full	Secondary atom site location: difference Fourier map
*R*[*F*^2^ > 2σ(*F*^2^)] = 0.051	Hydrogen site location: inferred from neighbouring sites
*wR*(*F*^2^) = 0.151	H-atom parameters constrained
*S* = 1.02	*w* = 1/[σ^2^(*F*_o_^2^) + (0.0678*P*)^2^ + 0.5852*P*] where *P* = (*F*_o_^2^ + 2*F*_c_^2^)/3
5086 reflections	(Δ/σ)_max_ < 0.001
273 parameters	Δρ_max_ = 0.35 e Å^−^^3^
0 restraints	Δρ_min_ = −0.39 e Å^−^^3^

### Special details

**Table:**

Geometry. All e.s.d.'s (except the e.s.d. in the dihedral angle between two l.s. planes) are estimated using the full covariance matrix. The cell e.s.d.'s are taken into account individually in the estimation of e.s.d.'s in distances, angles and torsion angles; correlations between e.s.d.'s in cell parameters are only used when they are defined by crystal symmetry. An approximate (isotropic) treatment of cell e.s.d.'s is used for estimating e.s.d.'s involving l.s. planes.
Refinement. Refinement of *F*^2^ against ALL reflections. The weighted *R*-factor *wR* and goodness of fit *S* are based on *F*^2^, conventional *R*-factors *R* are based on *F*, with *F* set to zero for negative *F*^2^. The threshold expression of *F*^2^ > σ(*F*^2^) is used only for calculating *R*-factors(gt) *etc*. and is not relevant to the choice of reflections for refinement. *R*-factors based on *F*^2^ are statistically about twice as large as those based on *F*, and *R*- factors based on ALL data will be even larger.

### Fractional atomic coordinates and isotropic or equivalent isotropic displacement parameters (Å^2^)

**Table:**

	*x*	*y*	*z*	*U*_iso_*/*U*_eq_	
C1	0.28173 (13)	0.2285 (2)	0.03324 (9)	0.0428 (4)	
C2	0.21242 (16)	0.2949 (3)	−0.03043 (10)	0.0549 (5)	
H2	0.1432	0.2921	−0.0385	0.066*	
C3	0.24774 (18)	0.3646 (3)	−0.08108 (10)	0.0641 (6)	
H3	0.2020	0.4092	−0.1236	0.077*	
C4	0.35030 (19)	0.3690 (3)	−0.06946 (10)	0.0610 (6)	
H4	0.3737	0.4181	−0.1036	0.073*	
C5	0.41795 (16)	0.3000 (2)	−0.00667 (10)	0.0499 (5)	
H5	0.4870	0.3013	0.0007	0.060*	
C6	0.38480 (13)	0.2284 (2)	0.04573 (9)	0.0396 (4)	
C7	0.46000 (12)	0.1483 (2)	0.11273 (8)	0.0362 (4)	
H7	0.4920	0.0503	0.0983	0.043*	
C8	0.41150 (12)	0.0860 (2)	0.16788 (8)	0.0354 (4)	
C9	0.31056 (13)	0.1770 (2)	0.15550 (9)	0.0433 (4)	
H9A	0.2788	0.1283	0.1874	0.052*	
H9B	0.3234	0.2971	0.1677	0.052*	
C10	0.49094 (12)	0.1477 (2)	0.24007 (8)	0.0363 (4)	
H10	0.5482	0.0676	0.2548	0.044*	
C11	0.64415 (12)	0.2269 (2)	0.16687 (9)	0.0366 (4)	
C12	0.67274 (15)	0.1419 (2)	0.11511 (10)	0.0486 (4)	
H12	0.6236	0.1026	0.0729	0.058*	
C13	0.77511 (17)	0.1158 (3)	0.12682 (13)	0.0592 (6)	
H13	0.7940	0.0584	0.0923	0.071*	
C14	0.84856 (16)	0.1735 (3)	0.18855 (13)	0.0594 (5)	
H14	0.9168	0.1567	0.1956	0.071*	
C15	0.82042 (14)	0.2561 (3)	0.23968 (12)	0.0544 (5)	
H15	0.8701	0.2944	0.2818	0.065*	
C16	0.71910 (13)	0.2836 (2)	0.22953 (10)	0.0435 (4)	
H16	0.7012	0.3401	0.2647	0.052*	
C17	0.45771 (12)	0.1822 (2)	0.30376 (8)	0.0387 (4)	
C18	0.43833 (14)	0.3468 (2)	0.32141 (10)	0.0469 (4)	
H18	0.4412	0.4378	0.2919	0.056*	
C19	0.41461 (14)	0.3771 (3)	0.38296 (10)	0.0529 (5)	
H19	0.4031	0.4887	0.3946	0.063*	
C20	0.40780 (14)	0.2438 (3)	0.42726 (10)	0.0526 (5)	
C21	0.42430 (16)	0.0803 (3)	0.40799 (10)	0.0591 (5)	
H21	0.4178	−0.0114	0.4361	0.071*	
C22	0.45034 (15)	0.0490 (3)	0.34767 (9)	0.0516 (5)	
H22	0.4630	−0.0626	0.3367	0.062*	
C23	0.3852 (2)	0.2806 (4)	0.49513 (12)	0.0751 (7)	
H23A	0.3697	0.1754	0.5142	0.113*	
H23B	0.3282	0.3567	0.4840	0.113*	
H23C	0.4433	0.3329	0.5303	0.113*	
C24	0.39583 (14)	−0.1064 (2)	0.16638 (9)	0.0438 (4)	
C25	0.3720 (2)	−0.3606 (3)	0.09983 (16)	0.0832 (8)	
H25A	0.4320	−0.4181	0.1303	0.125*	
H25B	0.3570	−0.3961	0.0507	0.125*	
H25C	0.3161	−0.3891	0.1149	0.125*	
N1	0.54050 (10)	0.27177 (18)	0.15286 (7)	0.0368 (3)	
O1	0.24239 (9)	0.16260 (17)	0.08243 (6)	0.0490 (3)	
O2	0.52327 (8)	0.30692 (14)	0.21877 (6)	0.0401 (3)	
O3	0.3903 (2)	−0.1874 (2)	0.21509 (9)	0.1145 (9)	
O4	0.38844 (14)	−0.17740 (17)	0.10527 (8)	0.0697 (5)	

### Atomic displacement parameters (Å^2^)

**Table:**

	*U*^11^	*U*^22^	*U*^33^	*U*^12^	*U*^13^	*U*^23^
C1	0.0519 (10)	0.0355 (9)	0.0363 (8)	−0.0049 (7)	0.0094 (7)	−0.0022 (7)
C2	0.0565 (11)	0.0533 (12)	0.0436 (10)	0.0008 (9)	0.0034 (8)	−0.0021 (8)
C3	0.0841 (16)	0.0579 (13)	0.0360 (9)	0.0009 (11)	0.0030 (9)	0.0054 (9)
C4	0.0884 (16)	0.0548 (13)	0.0381 (9)	−0.0088 (11)	0.0201 (10)	0.0060 (9)
C5	0.0642 (12)	0.0465 (11)	0.0398 (9)	−0.0085 (9)	0.0190 (8)	−0.0005 (8)
C6	0.0525 (10)	0.0304 (8)	0.0339 (8)	−0.0071 (7)	0.0126 (7)	−0.0040 (6)
C7	0.0435 (9)	0.0304 (8)	0.0351 (8)	−0.0050 (7)	0.0140 (7)	−0.0030 (6)
C8	0.0408 (8)	0.0336 (9)	0.0314 (7)	−0.0032 (7)	0.0123 (6)	−0.0007 (6)
C9	0.0420 (9)	0.0505 (11)	0.0367 (8)	−0.0014 (8)	0.0130 (7)	0.0019 (7)
C10	0.0375 (8)	0.0362 (9)	0.0354 (8)	−0.0007 (7)	0.0133 (6)	−0.0040 (7)
C11	0.0425 (9)	0.0313 (8)	0.0410 (8)	−0.0024 (7)	0.0208 (7)	0.0022 (7)
C12	0.0606 (11)	0.0467 (11)	0.0483 (10)	−0.0032 (9)	0.0312 (9)	−0.0022 (8)
C13	0.0742 (14)	0.0491 (12)	0.0769 (14)	0.0044 (10)	0.0549 (12)	0.0031 (10)
C14	0.0486 (11)	0.0531 (12)	0.0858 (15)	0.0058 (9)	0.0350 (11)	0.0145 (11)
C15	0.0433 (10)	0.0517 (12)	0.0670 (12)	−0.0033 (8)	0.0176 (9)	0.0066 (10)
C16	0.0437 (9)	0.0414 (10)	0.0479 (10)	−0.0033 (7)	0.0190 (7)	−0.0026 (8)
C17	0.0362 (8)	0.0455 (10)	0.0329 (8)	0.0014 (7)	0.0103 (6)	−0.0036 (7)
C18	0.0503 (10)	0.0484 (11)	0.0460 (10)	0.0016 (8)	0.0220 (8)	−0.0058 (8)
C19	0.0502 (10)	0.0604 (12)	0.0519 (11)	0.0054 (9)	0.0225 (9)	−0.0127 (9)
C20	0.0463 (10)	0.0751 (14)	0.0370 (9)	0.0064 (9)	0.0155 (7)	−0.0053 (9)
C21	0.0724 (13)	0.0688 (14)	0.0397 (10)	0.0044 (11)	0.0242 (9)	0.0074 (9)
C22	0.0664 (12)	0.0503 (11)	0.0390 (9)	0.0066 (9)	0.0196 (8)	0.0004 (8)
C23	0.0841 (16)	0.102 (2)	0.0469 (11)	0.0109 (14)	0.0328 (11)	−0.0068 (12)
C24	0.0516 (10)	0.0380 (9)	0.0379 (8)	−0.0074 (8)	0.0104 (7)	0.0017 (7)
C25	0.123 (2)	0.0343 (12)	0.106 (2)	−0.0138 (12)	0.0565 (18)	−0.0164 (12)
N1	0.0402 (7)	0.0371 (7)	0.0361 (7)	−0.0032 (6)	0.0169 (6)	−0.0053 (6)
O1	0.0406 (6)	0.0594 (8)	0.0419 (6)	−0.0064 (6)	0.0079 (5)	0.0042 (6)
O2	0.0450 (6)	0.0381 (7)	0.0427 (6)	−0.0064 (5)	0.0223 (5)	−0.0115 (5)
O3	0.230 (3)	0.0594 (11)	0.0599 (11)	−0.0586 (13)	0.0579 (14)	−0.0059 (8)
O4	0.1217 (13)	0.0332 (7)	0.0694 (10)	−0.0144 (8)	0.0526 (9)	−0.0117 (7)

### Geometric parameters (Å, º)

**Table:**

C1—O1	1.376 (2)	C13—C14	1.371 (3)
C1—C6	1.383 (2)	C13—H13	0.9300
C1—C2	1.395 (2)	C14—C15	1.369 (3)
C2—C3	1.376 (3)	C14—H14	0.9300
C2—H2	0.9300	C15—C16	1.385 (2)
C3—C4	1.380 (3)	C15—H15	0.9300
C3—H3	0.9300	C16—H16	0.9300
C4—C5	1.383 (3)	C17—C22	1.382 (3)
C4—H4	0.9300	C17—C18	1.384 (2)
C5—C6	1.393 (2)	C18—C19	1.390 (2)
C5—H5	0.9300	C18—H18	0.9300
C6—C7	1.512 (2)	C19—C20	1.385 (3)
C7—N1	1.488 (2)	C19—H19	0.9300
C7—C8	1.553 (2)	C20—C21	1.376 (3)
C7—H7	0.9800	C20—C23	1.511 (3)
C8—C24	1.518 (2)	C21—C22	1.386 (2)
C8—C9	1.529 (2)	C21—H21	0.9300
C8—C10	1.550 (2)	C22—H22	0.9300
C9—O1	1.432 (2)	C23—H23A	0.9600
C9—H9A	0.9700	C23—H23B	0.9600
C9—H9B	0.9700	C23—H23C	0.9600
C10—O2	1.4368 (19)	C24—O3	1.178 (2)
C10—C17	1.513 (2)	C24—O4	1.300 (2)
C10—H10	0.9800	C25—O4	1.447 (2)
C11—C16	1.389 (2)	C25—H25A	0.9600
C11—C12	1.392 (2)	C25—H25B	0.9600
C11—N1	1.429 (2)	C25—H25C	0.9600
C12—C13	1.391 (3)	N1—O2	1.4326 (16)
C12—H12	0.9300		
			
O1—C1—C6	121.96 (15)	C12—C13—H13	119.5
O1—C1—C2	116.73 (16)	C15—C14—C13	119.32 (18)
C6—C1—C2	121.31 (17)	C15—C14—H14	120.3
C3—C2—C1	119.17 (19)	C13—C14—H14	120.3
C3—C2—H2	120.4	C14—C15—C16	121.0 (2)
C1—C2—H2	120.4	C14—C15—H15	119.5
C2—C3—C4	120.76 (18)	C16—C15—H15	119.5
C2—C3—H3	119.6	C15—C16—C11	120.13 (17)
C4—C3—H3	119.6	C15—C16—H16	119.9
C3—C4—C5	119.41 (19)	C11—C16—H16	119.9
C3—C4—H4	120.3	C22—C17—C18	118.46 (16)
C5—C4—H4	120.3	C22—C17—C10	120.09 (16)
C4—C5—C6	121.32 (19)	C18—C17—C10	121.39 (15)
C4—C5—H5	119.3	C17—C18—C19	120.50 (18)
C6—C5—H5	119.3	C17—C18—H18	119.7
C1—C6—C5	118.01 (16)	C19—C18—H18	119.7
C1—C6—C7	121.70 (15)	C20—C19—C18	121.11 (19)
C5—C6—C7	120.26 (16)	C20—C19—H19	119.4
N1—C7—C6	111.58 (13)	C18—C19—H19	119.4
N1—C7—C8	105.54 (12)	C21—C20—C19	117.84 (17)
C6—C7—C8	113.59 (13)	C21—C20—C23	122.1 (2)
N1—C7—H7	108.7	C19—C20—C23	120.1 (2)
C6—C7—H7	108.7	C20—C21—C22	121.52 (19)
C8—C7—H7	108.7	C20—C21—H21	119.2
C24—C8—C9	109.67 (14)	C22—C21—H21	119.2
C24—C8—C10	112.27 (13)	C17—C22—C21	120.52 (19)
C9—C8—C10	109.47 (13)	C17—C22—H22	119.7
C24—C8—C7	113.13 (13)	C21—C22—H22	119.7
C9—C8—C7	110.69 (13)	C20—C23—H23A	109.5
C10—C8—C7	101.35 (12)	C20—C23—H23B	109.5
O1—C9—C8	112.46 (14)	H23A—C23—H23B	109.5
O1—C9—H9A	109.1	C20—C23—H23C	109.5
C8—C9—H9A	109.1	H23A—C23—H23C	109.5
O1—C9—H9B	109.1	H23B—C23—H23C	109.5
C8—C9—H9B	109.1	O3—C24—O4	121.62 (18)
H9A—C9—H9B	107.8	O3—C24—C8	124.52 (17)
O2—C10—C17	108.13 (13)	O4—C24—C8	113.86 (15)
O2—C10—C8	101.51 (12)	O4—C25—H25A	109.5
C17—C10—C8	119.25 (13)	O4—C25—H25B	109.5
O2—C10—H10	109.1	H25A—C25—H25B	109.5
C17—C10—H10	109.1	O4—C25—H25C	109.5
C8—C10—H10	109.1	H25A—C25—H25C	109.5
C16—C11—C12	118.91 (16)	H25B—C25—H25C	109.5
C16—C11—N1	119.98 (14)	C11—N1—O2	110.86 (12)
C12—C11—N1	120.78 (15)	C11—N1—C7	118.62 (13)
C13—C12—C11	119.71 (18)	O2—N1—C7	105.67 (11)
C13—C12—H12	120.1	C1—O1—C9	112.99 (13)
C11—C12—H12	120.1	N1—O2—C10	105.79 (11)
C14—C13—C12	120.97 (18)	C24—O4—C25	116.71 (17)
C14—C13—H13	119.5		
			
O1—C1—C2—C3	179.09 (17)	O2—C10—C17—C22	−161.69 (15)
C6—C1—C2—C3	−1.3 (3)	C8—C10—C17—C22	83.2 (2)
C1—C2—C3—C4	0.1 (3)	O2—C10—C17—C18	15.5 (2)
C2—C3—C4—C5	1.1 (3)	C8—C10—C17—C18	−99.61 (19)
C3—C4—C5—C6	−1.1 (3)	C22—C17—C18—C19	1.6 (3)
O1—C1—C6—C5	−179.12 (16)	C10—C17—C18—C19	−175.59 (15)
C2—C1—C6—C5	1.2 (3)	C17—C18—C19—C20	−1.3 (3)
O1—C1—C6—C7	3.0 (3)	C18—C19—C20—C21	−0.6 (3)
C2—C1—C6—C7	−176.68 (16)	C18—C19—C20—C23	178.06 (19)
C4—C5—C6—C1	−0.1 (3)	C19—C20—C21—C22	2.2 (3)
C4—C5—C6—C7	177.88 (17)	C23—C20—C21—C22	−176.5 (2)
C1—C6—C7—N1	−125.26 (16)	C18—C17—C22—C21	−0.1 (3)
C5—C6—C7—N1	56.9 (2)	C10—C17—C22—C21	177.14 (17)
C1—C6—C7—C8	−6.1 (2)	C20—C21—C22—C17	−1.8 (3)
C5—C6—C7—C8	176.00 (15)	C9—C8—C24—O3	−80.1 (3)
N1—C7—C8—C24	−134.90 (14)	C10—C8—C24—O3	41.9 (3)
C6—C7—C8—C24	102.56 (16)	C7—C8—C24—O3	155.8 (2)
N1—C7—C8—C9	101.55 (15)	C9—C8—C24—O4	99.69 (18)
C6—C7—C8—C9	−20.99 (18)	C10—C8—C24—O4	−138.38 (16)
N1—C7—C8—C10	−14.50 (15)	C7—C8—C24—O4	−24.4 (2)
C6—C7—C8—C10	−137.04 (14)	C16—C11—N1—O2	−25.1 (2)
C24—C8—C9—O1	−72.08 (17)	C12—C11—N1—O2	161.65 (14)
C10—C8—C9—O1	164.32 (13)	C16—C11—N1—C7	−147.58 (15)
C7—C8—C9—O1	53.43 (18)	C12—C11—N1—C7	39.2 (2)
C24—C8—C10—O2	156.94 (13)	C6—C7—N1—C11	−123.39 (15)
C9—C8—C10—O2	−81.01 (14)	C8—C7—N1—C11	112.79 (15)
C7—C8—C10—O2	35.94 (14)	C6—C7—N1—O2	111.56 (14)
C24—C8—C10—C17	−84.50 (19)	C8—C7—N1—O2	−12.26 (15)
C9—C8—C10—C17	37.5 (2)	C6—C1—O1—C9	29.8 (2)
C7—C8—C10—C17	154.50 (15)	C2—C1—O1—C9	−150.52 (16)
C16—C11—C12—C13	−0.2 (3)	C8—C9—O1—C1	−58.64 (19)
N1—C11—C12—C13	173.12 (16)	C11—N1—O2—C10	−92.54 (14)
C11—C12—C13—C14	−0.3 (3)	C7—N1—O2—C10	37.19 (14)
C12—C13—C14—C15	0.8 (3)	C17—C10—O2—N1	−172.31 (12)
C13—C14—C15—C16	−0.7 (3)	C8—C10—O2—N1	−46.05 (13)
C14—C15—C16—C11	0.1 (3)	O3—C24—O4—C25	0.6 (3)
C12—C11—C16—C15	0.3 (3)	C8—C24—O4—C25	−179.18 (19)
N1—C11—C16—C15	−173.06 (16)		

### Hydrogen-bond geometry (Å, º)

Cg3 and Cg5 are the centroids of rings C1–C6 and C17–C22, respectively.

**Table:**

*D*—H···*A*	*D*—H	H···*A*	*D*···*A*	*D*—H···*A*
C2—H2···*Cg*5^i^	0.93	2.91	3.757 (2)	151
C25—H25*B*···*Cg*3^ii^	0.96	2.71	3.450 (3)	134

Symmetry codes: (i) *x*−1/2, −*y*+1/2, *z*−1/2; (ii) *x*, *y*−1, *z*.
